# Spongoid

**Published:** 1874-11

**Authors:** 


*Spongoid.*—In the *Dental Miscellany* Dr. J. A. Kimball, notices this new absorbent as follows:

"Not having seen any reference to this article, recently introduced into the department of operative dentistry, I take the liberty of calling attention to it. Having employed it constantly for the past three months, I am of the opinion that it is of sufficient merit to deserve a friendly recognition at the hands of the dental profession.

To those who have had no opportunity of examining it, a brief description may be interesting. The article is designed, so far as its application to dentistry is concerned, to supply the place of prepared cotton, spunk and bibulous paper in drying ordinary cavities. In appearance it resembles white blotting paper, and the substances of the two articles are doubtless quite similar; but the spongoid is more fibrous and much less compact, owing, of course, to a different quality of material and a different treatment in the process of manufaeture. And even common blotting paper is not a contemptible absorbent for dental purposes, as I learned years ago while treating a tooth for a little patient in the country, without the usual accessories of the office.

The spongoid is prepared in three forms: first, in small round discs; second, in long strips about one-fourth of an inch wide, and third, in square sheets nearly the size of a sheet of gold foil. I prefer the latter form for ordinary use, as it can easily be torn into pieces of any required size and of sufficient softness to be pressed into all the inequalities of the cavity to be dried. The three forms are of various thicknesses, to enable them to meet all cases. The discs are of different sizes, and possess the advantage of being always ready, while the strips give less trouble than the sheets in reducing them to pellets of suitable dimensions; yet the process of cutting so condenses the edges of the discs and strips as to greatly lessen their adaptability, and for this reason, principally, I choose the sheets.

The absorbing power of this substance is marvelous. Place a drop of water upon a slab and approach it gradually with one of the discs. Upon the slighest contact the drop instantly disappears, and the disc becomes uniformly charged with the moisture. Dip one end of a strip of spongoid in a glass of water and almost immediately the fluid ascends to its entire extent and the strip is completely saturated. Nothing but the best of marine sponge can equal the spongoid in its absorbent properties.

Regarding its practical employment, I see no reason why the spongoid should not supersede, in ordinary cases, all substances heretofore used. It cannot, however, supply the place of cotton in drying pulp cavities, nor that of the arch detective spunk in discovering the presence of slight moisture. I venture no extravagant predictions concerning the spongoid, but I dare say it will, sooner or later, of its own intrinsic merit, assume its proper and natural place in the dentist's repertoire, and become a valuable adjunct to his operations.

It is, doubtless, in surgery that this preparation will find its most important and appropriate work. For this department it is medicated so as to act as a styptic, an antiseptic, a counter irritant and vesicant, an anodyne or a disinfectant, as well as an absorbent. It is needless to suggest that these medicated forms may be of occasional use in our own profession. I have myself employed the styptic spongoid, and find it admirable. It is less unpleasant and far more convenient and cleanly than Rohland's styptic cotton, and equally effective as an article of ready application. There are sheets expressly prepared for large suppurating surfaces by being thickly perforated. The sheet itself, which has been treated with carbolic acid, or some other antiseptic, absorbs and neutralizes the serous portion of the effusion, while the more concrete portion escapes through the small openings, and is removed when the sheet is detached. An eminent surgeon of this city assures me he finds it of great value in this class of cases, and it has been successfully employed in Europe for similar purposes.

In conclusion, I may perhaps be permitted to say, incidentally, that in my practice I use absorbents but little. Where there is danger of the cavity becoming wet in excavating, I usually attach the Rubber Dam, and it is then in place when I am ready to insert the filling. The dentine is less sensitive if excavated dry, and the debris of excavation does not require the syringe, as it may be most thoroughly removed by a bit of cotton or spongoid slightly dampened with chloroform. That the latter agent in evaporation leaves no moisture behind, I have repeatedly proved in cases where the gold became accidently wet during the process of filling. After adopting means to prevent any further access of saliva, I have bathed the surface of the interrupted plug with chloroform, and when the volatile fluid had escaped, proceeded with the operation, the gold adhering with almost, if not quite, the same tenacity as if nothing unfavorable had occurred."

